# 4-Amino­pyridinium 4-nitro­benzoate 4-nitro­benzoic acid

**DOI:** 10.1107/S1600536808027761

**Published:** 2008-09-06

**Authors:** Ching Kheng Quah, Samuel Robinson Jebas, Hoong-Kun Fun

**Affiliations:** a X-ray Crystallography Unit, School of Physics, Universiti Sains Malaysia, 11800 USM, Penang, Malaysia

## Abstract

The asymmetric unit of the title compound, C_5_H_7_N_2_^+^·C_7_H_4_NO_4_^−^·C_7_H_5_NO_4_, consists of an amino­pyridinium cation, a 4-nitro­benzoate anion and a neutral 4-nitro­benzoic acid mol­ecule. The pyridine ring forms dihedral angles of 64.70 (5)° and 70.37 (5)°, respectively, with the benzene rings of 4-nitro­benzoic acid and 4-nitro­benzoate. In the crystal structure, the cations, anions and the neutral 4-nitro­benzoic acid mol­ecules are linked by O—H⋯O and N—H⋯O hydrogen bonds, forming a two-dimensional network parallel to (001). Adjacent networks are cross-linked *via* C—H⋯O hydrogen bonds and π–π stacking inter­actions [centroid–centroid distances 3.6339 (6) and 3.6566 (6) Å].

## Related literature

For the biological activity of 4-amino­pyridine, see: Judge *et al.* (2006 ▶); Schwid *et al.* (1997 ▶); Strupp *et al.* (2004 ▶). For related structures, see: Chao & Schempp (1977 ▶); Anderson *et al.* (2005 ▶); Andrau & White, (2003 ▶); Bhattacharya *et al.* (1994 ▶); Karle *et al.* (2003 ▶).
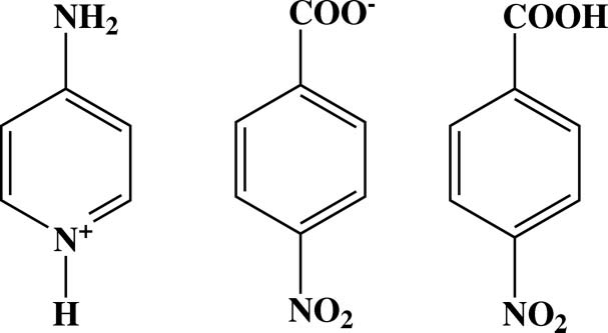


## Experimental

### 

#### Crystal data

C_5_H_7_N_2_^+^·C_7_H_4_NO_4_^−^·C_7_H_5_NO_4_
*M* *_r_* = 428.36
Triclinic, 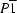

*a* = 6.4561 (1) Å
*b* = 6.8598 (1) Å
*c* = 20.9055 (3) Å
α = 85.826 (1)°
β = 87.975 (1)°
γ = 86.188 (1)°
*V* = 920.92 (2) Å^3^
*Z* = 2
Mo *K*α radiation
μ = 0.12 mm^−1^
*T* = 100.0 (1) K
0.40 × 0.36 × 0.29 mm

#### Data collection

Bruker SMART APEXII CCD area-detector diffractometer
Absorption correction: multi-scan (*SADABS*; Bruker, 2005 ▶) *T*_min_ = 0.952, *T*_max_ = 0.965
24945 measured reflections
6647 independent reflections
5169 reflections with *I* > 2σ(*I*)
*R*_int_ = 0.031

#### Refinement

*R*[*F* ^2^ > 2σ(*F* ^2^)] = 0.044
*wR*(*F* ^2^) = 0.132
*S* = 1.05
6647 reflections
284 parameters
1 restraint
H atoms treated by a mixture of independent and constrained refinement
Δρ_max_ = 0.43 e Å^−3^
Δρ_min_ = −0.39 e Å^−3^

### 

Data collection: *APEX2* (Bruker, 2005 ▶); cell refinement: *APEX2*; data reduction: *SAINT* (Bruker, 2005 ▶); program(s) used to solve structure: *SHELXTL* (Sheldrick, 2008 ▶); program(s) used to refine structure: *SHELXTL*; molecular graphics: *SHELXTL*; software used to prepare material for publication: *SHELXTL* and *PLATON* (Spek, 2003 ▶).

## Supplementary Material

Crystal structure: contains datablocks global, I. DOI: 10.1107/S1600536808027761/ci2664sup1.cif

Structure factors: contains datablocks I. DOI: 10.1107/S1600536808027761/ci2664Isup2.hkl

Additional supplementary materials:  crystallographic information; 3D view; checkCIF report

## Figures and Tables

**Table 1:** Hydrogen-bond geometry (Å, °)

*D*—H⋯*A*	*D*—H	H⋯*A*	*D*⋯*A*	*D*—H⋯*A*
O3*A*—H1*O*3⋯O3*B*^i^	0.82	1.63	2.4457 (11)	170
N3—H3*A*⋯O3*B*^ii^	0.86	2.14	2.9977 (12)	172
N3—H3*B*⋯O4*B*^i^	0.86	2.07	2.8758 (12)	155
N2—H1*N*2⋯O4*A*^iii^	0.85 (1)	1.99 (1)	2.7726 (12)	153 (1)
C2*B*—H2*BA*⋯O1*B*^iv^	0.93	2.52	3.2187 (13)	133
C8—H8*A*⋯O3*A*^v^	0.93	2.56	3.4565 (13)	161
C12—H12*A*⋯O1*A*^vi^	0.93	2.55	3.4427 (13)	162

Symmetry codes: (i) 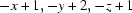; (ii) 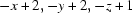; (iii) 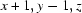; (iv) 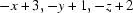; (v) 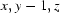; (vi) 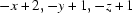.

**Fig. 1.**
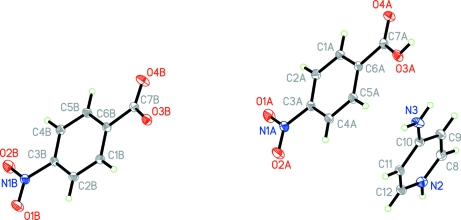
The asymmetric unit of the title compound, showing 50% probability displacement ellipsoids and the atom-numbering scheme.

**Fig. 2.**
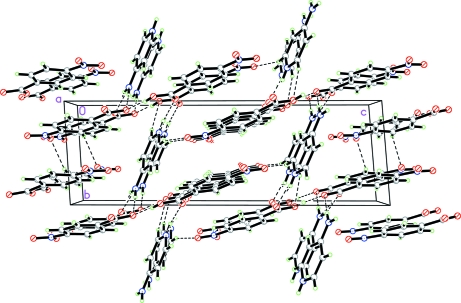
The crystal packing of the title compound, viewed along the a axis. Hydrogen bonds are shown as dashed lines.

## supplementary crystallographic information

### Comment

4-Aminopyridine (Fampridine) is used clinically in Lambert-Eaton myasthenic syndrome and multiple sclerosis because by blocking potassium channels, it prolongs the action potentials thereby increasing transmitter release at the neuromuscular junction (Judge *et al.*, 2006; Schwid *et al.*, 1997; Strupp *et al.*, 2004). The crystal structure of 4-aminopyridine has been reported (Chao & Schempp, 1977; Anderson *et al.*, 2005). As an extension of our systematic study of hydrogen bonding patterns of 4-aminopyridine with aromatic carboxylic acids, we report here the crystal structure of the title compound.

The asymmetric unit of the title compound contains one 4-aminopyridinium cation, one 4-nitrobenzoate anion and one 4-nitrobenzoic acid molecule. A proton transfer from the carboxyl group of 4-nitrobenzoic acid to atom N2 of 4-aminopyridine resulted in the formation of ions. This lead to the widening of C8—N2—C12 angle of the pyridine ring to 120.86 (9)°, compared to 115.25 (13)° in the unprotonated 4-aminopyridine (Anderson *et al.*, 2005). This type of protonation is observed in various 4-aminopyridine acid complexes (Bhattacharya *et al.*, 1994; Karle *et al.*, 2003). The bond lengths and angles of the 4-aminopyridne are comparable to the values reported earlier for 4-aminopyridine (Chao & Schempp, 1977; Anderson *et al.*, 2005). The bond lengths and angles of the 4-nitrobenzoic acid is found to be normal(Andrau & White, 2003).

The dihedral angle between the benzene rings of 4-nitrobenzoic acid (C1A-C6A) and 4-nitrobenzoate (C1B-C6B) units is 6.62 (5)°. The pyridine (N2/C8—C12) ring forms dihedral angles of 64.70 (5)° and 70.37 (5)°, respectively, with the C1A-C6A and C1B-C6B rings.

In the crystal structure, the cations, anions and the neutral 4-nitrobenzoic acid molecules are linked to form a two-dimensional network (Fig. 2) parallel to the (0 0 1) by O—H···O and N—H···O hydrogen bonds (Table 1). The adjacent networks are cross-linked via C—H···O hydrogen bonds. The crystal packing is further consolidated by π–π stacking interactions between symmetry-related C1A-C6A (centroid *Cg*1) and C1B-C6B (centroid *Cg*2) rings, with *Cg*1···*Cg*1^i^ and *Cg*2···*Cg*2^vii^ distances of 3.6566 (6) Å and 3.6339 (6) Å, respectively [symmetry codes: (i) 1-x, 2-y, 1-z; (vii) 2-x, 2-y, 2-z].

### Experimental

4-Aminopyridine and 4-nitrobenzoic acid were mixed in equimolar ratio in methanol and warmed in a water bath for 2 h. Colourless single crystals were obtained after a week on slow evaporation.

### Refinement

Atom H1N2 was located from a difference map and was refined with the N-H distance restrained to 0.85 (1) Å. The remaining H atoms were positioned geometrically with C-H = 0.93 Å, N-H = 0.86 Å and O-H = 0.82Å, and refined using a riding model, with *U*_iso_(H) = 1.2*U*_eq_(C,N) and 1.5*U*_eq_(O).

### Crystal data

**Table:**

C_5_H_7_N_2_^+^·C_7_H_4_NO_4_^−^·C_7_H_5_NO_4_	*Z* = 2
*M**_r_* = 428.36	*F*(000) = 444
Triclinic, *P*1	*D*_x_ = 1.545 Mg m^−^^3^
Hall symbol: -P 1	Mo *K*α radiation, λ = 0.71073 Å
*a* = 6.4561 (1) Å	Cell parameters from 6200 reflections
*b* = 6.8598 (1) Å	θ = 2.2–29.2°
*c* = 20.9055 (3) Å	µ = 0.12 mm^−^^1^
α = 85.826 (1)°	*T* = 100 K
β = 87.975 (1)°	Block, colourless
γ = 86.188 (1)°	0.40 × 0.36 × 0.29 mm
*V* = 920.92 (2) Å^3^	

### Data collection

**Table:**

Bruker SMART APEXII CCD area-detector diffractometer	6647 independent reflections
Radiation source: fine-focus sealed tube	5169 reflections with *I* > 2σ(*I*)
graphite	*R*_int_ = 0.031
φ and ω scans	θ_max_ = 32.5°, θ_min_ = 1.0°
Absorption correction: multi-scan (SADABS; Bruker, 2005)	*h* = −9→9
*T*_min_ = 0.952, *T*_max_ = 0.965	*k* = −10→10
24945 measured reflections	*l* = −31→31

### Refinement

**Table:**

Refinement on *F*^2^	Primary atom site location: structure-invariant direct methods
Least-squares matrix: full	Secondary atom site location: difference Fourier map
*R*[*F*^2^ > 2σ(*F*^2^)] = 0.044	Hydrogen site location: inferred from neighbouring sites
*wR*(*F*^2^) = 0.132	H atoms treated by a mixture of independent and constrained refinement
*S* = 1.05	*w* = 1/[σ^2^(*F*_o_^2^) + (0.0736*P*)^2^ + 0.1221*P*] where *P* = (*F*_o_^2^ + 2*F*_c_^2^)/3
6647 reflections	(Δ/σ)_max_ = 0.001
284 parameters	Δρ_max_ = 0.43 e Å^−^^3^
1 restraint	Δρ_min_ = −0.39 e Å^−^^3^

### Special details

**Table:**

Experimental. The data was collected with the Oxford Cyrosystem Cobra low-temperature attachment.
Geometry. All e.s.d.'s (except the e.s.d. in the dihedral angle between two l.s. planes) are estimated using the full covariance matrix. The cell e.s.d.'s are taken into account individually in the estimation of e.s.d.'s in distances, angles and torsion angles; correlations between e.s.d.'s in cell parameters are only used when they are defined by crystal symmetry. An approximate (isotropic) treatment of cell e.s.d.'s is used for estimating e.s.d.'s involving l.s. planes.
Refinement. Refinement of *F*^2^ against ALL reflections. The weighted *R*-factor *wR* and goodness of fit *S* are based on *F*^2^, conventional *R*-factors *R* are based on *F*, with *F* set to zero for negative *F*^2^. The threshold expression of *F*^2^ > σ(*F*^2^) is used only for calculating *R*-factors(gt) *etc*. and is not relevant to the choice of reflections for refinement. *R*-factors based on *F*^2^ are statistically about twice as large as those based on *F*, and *R*- factors based on ALL data will be even larger.

### Fractional atomic coordinates and isotropic or equivalent isotropic displacement parameters (Å^2^)

**Table:**

	*x*	*y*	*z*	*U*_iso_*/*U*_eq_	
O1A	0.61416 (13)	0.64153 (13)	0.61139 (4)	0.02534 (18)	
O1B	1.43798 (12)	0.64460 (14)	1.07244 (4)	0.02613 (19)	
O2A	0.89023 (12)	0.63198 (13)	0.54960 (4)	0.02492 (18)	
O2B	1.15700 (13)	0.66730 (15)	1.13139 (4)	0.02794 (19)	
O3A	0.31404 (11)	0.97209 (12)	0.29069 (4)	0.02024 (16)	
H1O3	0.2342	1.0163	0.2628	0.030*	
O3B	0.89111 (11)	0.90736 (11)	0.80183 (4)	0.01831 (15)	
O4A	0.02402 (11)	0.98398 (11)	0.35318 (4)	0.01950 (16)	
O4B	0.59084 (12)	0.90329 (13)	0.85892 (4)	0.02425 (18)	
N1A	0.70215 (13)	0.66162 (13)	0.55851 (4)	0.01667 (17)	
N1B	1.24848 (13)	0.67136 (13)	1.07904 (4)	0.01646 (17)	
N2	0.80980 (14)	0.30341 (13)	0.29153 (4)	0.01904 (18)	
N3	0.73597 (13)	0.85350 (13)	0.20200 (5)	0.02020 (18)	
H3A	0.8354	0.9309	0.2022	0.024*	
H3B	0.6223	0.8922	0.1834	0.024*	
C1A	0.24956 (15)	0.84572 (14)	0.46101 (5)	0.01507 (18)	
H1AA	0.1084	0.8790	0.4667	0.018*	
C1B	1.11431 (15)	0.75402 (14)	0.90711 (5)	0.01505 (18)	
H1BA	1.1788	0.7482	0.8667	0.018*	
C2A	0.36551 (15)	0.77640 (14)	0.51351 (5)	0.01593 (18)	
H2AA	0.3048	0.7633	0.5545	0.019*	
C2B	1.22778 (15)	0.70459 (14)	0.96180 (5)	0.01522 (18)	
H2BA	1.3685	0.6664	0.9587	0.018*	
C3B	1.12614 (14)	0.71350 (14)	1.02109 (5)	0.01397 (17)	
C3A	0.57532 (15)	0.72731 (14)	0.50276 (5)	0.01424 (17)	
C4A	0.67164 (15)	0.74110 (15)	0.44250 (5)	0.01592 (18)	
H4AA	0.8119	0.7038	0.4369	0.019*	
C4B	0.91589 (15)	0.76542 (14)	1.02843 (5)	0.01527 (18)	
H4BA	0.8513	0.7667	1.0689	0.018*	
C5A	0.55303 (15)	0.81214 (15)	0.39071 (5)	0.01621 (18)	
H5AA	0.6142	0.8239	0.3498	0.019*	
C5B	0.80500 (15)	0.81538 (14)	0.97324 (5)	0.01517 (18)	
H5BA	0.6638	0.8512	0.9766	0.018*	
C6A	0.34218 (14)	0.86598 (14)	0.39989 (5)	0.01412 (17)	
C6B	0.90370 (14)	0.81237 (14)	0.91270 (5)	0.01388 (17)	
C7A	0.21250 (15)	0.94696 (14)	0.34447 (5)	0.01498 (18)	
C7B	0.78049 (15)	0.87820 (14)	0.85437 (5)	0.01578 (18)	
C8	0.62995 (16)	0.35854 (16)	0.26207 (5)	0.0195 (2)	
H8A	0.5260	0.2709	0.2623	0.023*	
C9	0.59889 (15)	0.54066 (15)	0.23204 (5)	0.01760 (19)	
H9A	0.4736	0.5775	0.2126	0.021*	
C10	0.75805 (15)	0.67391 (15)	0.23052 (5)	0.01566 (18)	
C11	0.94501 (15)	0.60896 (15)	0.26128 (5)	0.01670 (19)	
H11A	1.0538	0.6914	0.2611	0.020*	
C12	0.96496 (16)	0.42621 (16)	0.29097 (5)	0.0186 (2)	
H12A	1.0877	0.3849	0.3113	0.022*	
H1N2	0.839 (2)	0.1892 (15)	0.3080 (7)	0.029 (4)*	

### Atomic displacement parameters (Å^2^)

**Table:**

	*U*^11^	*U*^22^	*U*^33^	*U*^12^	*U*^13^	*U*^23^
O1A	0.0250 (4)	0.0384 (5)	0.0118 (4)	−0.0024 (3)	−0.0008 (3)	0.0048 (3)
O1B	0.0158 (3)	0.0428 (5)	0.0191 (4)	0.0041 (3)	−0.0028 (3)	−0.0013 (3)
O2A	0.0172 (3)	0.0361 (5)	0.0207 (4)	0.0016 (3)	−0.0036 (3)	0.0012 (3)
O2B	0.0228 (4)	0.0485 (5)	0.0115 (4)	0.0012 (3)	0.0006 (3)	0.0003 (3)
O3A	0.0183 (3)	0.0303 (4)	0.0116 (3)	−0.0013 (3)	−0.0025 (3)	0.0027 (3)
O3B	0.0174 (3)	0.0258 (4)	0.0114 (3)	−0.0009 (3)	−0.0003 (3)	0.0006 (3)
O4A	0.0158 (3)	0.0236 (4)	0.0182 (4)	0.0011 (3)	−0.0016 (3)	0.0026 (3)
O4B	0.0145 (3)	0.0368 (5)	0.0198 (4)	0.0011 (3)	−0.0020 (3)	0.0071 (3)
N1A	0.0179 (4)	0.0178 (4)	0.0145 (4)	−0.0016 (3)	−0.0034 (3)	−0.0001 (3)
N1B	0.0167 (4)	0.0194 (4)	0.0131 (4)	0.0003 (3)	−0.0014 (3)	−0.0010 (3)
N2	0.0213 (4)	0.0188 (4)	0.0161 (4)	0.0014 (3)	0.0009 (3)	0.0017 (3)
N3	0.0160 (4)	0.0212 (4)	0.0224 (5)	0.0005 (3)	−0.0014 (3)	0.0045 (3)
C1A	0.0146 (4)	0.0166 (4)	0.0140 (4)	−0.0011 (3)	−0.0003 (3)	−0.0006 (3)
C1B	0.0156 (4)	0.0177 (4)	0.0114 (4)	0.0009 (3)	0.0001 (3)	−0.0004 (3)
C2A	0.0172 (4)	0.0180 (4)	0.0127 (4)	−0.0030 (3)	0.0005 (3)	−0.0001 (3)
C2B	0.0139 (4)	0.0175 (4)	0.0140 (4)	0.0009 (3)	−0.0001 (3)	−0.0010 (3)
C3B	0.0151 (4)	0.0154 (4)	0.0114 (4)	−0.0001 (3)	−0.0023 (3)	−0.0003 (3)
C3A	0.0168 (4)	0.0143 (4)	0.0118 (4)	−0.0017 (3)	−0.0030 (3)	0.0002 (3)
C4A	0.0141 (4)	0.0193 (4)	0.0143 (4)	−0.0007 (3)	−0.0005 (3)	−0.0013 (3)
C4B	0.0158 (4)	0.0175 (4)	0.0125 (4)	−0.0018 (3)	0.0009 (3)	−0.0007 (3)
C5A	0.0163 (4)	0.0206 (4)	0.0117 (4)	−0.0015 (3)	0.0000 (3)	−0.0013 (3)
C5B	0.0131 (4)	0.0180 (4)	0.0142 (4)	−0.0005 (3)	−0.0002 (3)	0.0001 (3)
C6A	0.0154 (4)	0.0147 (4)	0.0125 (4)	−0.0018 (3)	−0.0021 (3)	−0.0006 (3)
C6B	0.0143 (4)	0.0151 (4)	0.0122 (4)	−0.0013 (3)	−0.0014 (3)	0.0005 (3)
C7A	0.0173 (4)	0.0153 (4)	0.0125 (4)	−0.0026 (3)	−0.0018 (3)	0.0002 (3)
C7B	0.0157 (4)	0.0167 (4)	0.0149 (4)	−0.0007 (3)	−0.0022 (3)	−0.0001 (3)
C8	0.0175 (4)	0.0233 (5)	0.0178 (5)	−0.0023 (4)	0.0008 (4)	−0.0016 (4)
C9	0.0138 (4)	0.0231 (5)	0.0156 (5)	−0.0006 (3)	−0.0012 (3)	−0.0001 (4)
C10	0.0144 (4)	0.0199 (4)	0.0123 (4)	0.0012 (3)	0.0005 (3)	−0.0009 (3)
C11	0.0151 (4)	0.0203 (4)	0.0148 (4)	−0.0004 (3)	−0.0020 (3)	−0.0015 (4)
C12	0.0178 (4)	0.0232 (5)	0.0143 (5)	0.0029 (3)	−0.0023 (3)	−0.0010 (4)

### Geometric parameters (Å, °)

**Table:**

O1A—N1A	1.2278 (12)	C2A—H2AA	0.93
O1B—N1B	1.2294 (11)	C2B—C3B	1.3851 (14)
O2A—N1A	1.2276 (11)	C2B—H2BA	0.93
O2B—N1B	1.2244 (12)	C3B—C4B	1.3863 (13)
O3A—C7A	1.2877 (12)	C3A—C4A	1.3848 (14)
O3A—H1O3	0.8200	C4A—C5A	1.3888 (14)
O3B—C7B	1.2993 (12)	C4A—H4AA	0.93
O4A—C7A	1.2362 (12)	C4B—C5B	1.3890 (14)
O4B—C7B	1.2263 (12)	C4B—H4BA	0.93
N1A—C3A	1.4743 (12)	C5A—C6A	1.3969 (13)
N1B—C3B	1.4702 (13)	C5A—H5AA	0.93
N2—C12	1.3502 (14)	C5B—C6B	1.3977 (14)
N2—C8	1.3523 (14)	C5B—H5BA	0.93
N2—H1N2	0.844 (9)	C6A—C7A	1.5049 (13)
N3—C10	1.3301 (13)	C6B—C7B	1.5047 (13)
N3—H3A	0.86	C8—C9	1.3626 (15)
N3—H3B	0.86	C8—H8A	0.93
C1A—C2A	1.3877 (14)	C9—C10	1.4180 (14)
C1A—C6A	1.3930 (14)	C9—H9A	0.93
C1A—H1AA	0.93	C10—C11	1.4180 (13)
C1B—C2B	1.3888 (13)	C11—C12	1.3580 (15)
C1B—C6B	1.3949 (13)	C11—H11A	0.93
C1B—H1BA	0.93	C12—H12A	0.93
C2A—C3A	1.3884 (13)		
			
C7A—O3A—H1O3	109.5	C3B—C4B—H4BA	121.2
O2A—N1A—O1A	123.62 (9)	C5B—C4B—H4BA	121.2
O2A—N1A—C3A	118.18 (9)	C4A—C5A—C6A	120.21 (9)
O1A—N1A—C3A	118.20 (8)	C4A—C5A—H5AA	119.9
O2B—N1B—O1B	123.36 (9)	C6A—C5A—H5AA	119.9
O2B—N1B—C3B	118.43 (8)	C4B—C5B—C6B	120.61 (9)
O1B—N1B—C3B	118.20 (9)	C4B—C5B—H5BA	119.7
C12—N2—C8	120.86 (9)	C6B—C5B—H5BA	119.7
C12—N2—H1N2	115.2 (11)	C1A—C6A—C5A	119.99 (9)
C8—N2—H1N2	123.7 (11)	C1A—C6A—C7A	119.15 (8)
C10—N3—H3A	120.0	C5A—C6A—C7A	120.86 (9)
C10—N3—H3B	120.0	C1B—C6B—C5B	120.08 (9)
H3A—N3—H3B	120.0	C1B—C6B—C7B	121.02 (9)
C2A—C1A—C6A	120.74 (9)	C5B—C6B—C7B	118.88 (8)
C2A—C1A—H1AA	119.6	O4A—C7A—O3A	125.63 (9)
C6A—C1A—H1AA	119.6	O4A—C7A—C6A	119.65 (9)
C2B—C1B—C6B	120.04 (9)	O3A—C7A—C6A	114.72 (8)
C2B—C1B—H1BA	120.0	O4B—C7B—O3B	125.05 (9)
C6B—C1B—H1BA	120.0	O4B—C7B—C6B	120.18 (9)
C1A—C2A—C3A	117.70 (9)	O3B—C7B—C6B	114.75 (8)
C1A—C2A—H2AA	121.2	N2—C8—C9	120.94 (10)
C3A—C2A—H2AA	121.2	N2—C8—H8A	119.5
C3B—C2B—C1B	118.35 (9)	C9—C8—H8A	119.5
C3B—C2B—H2BA	120.8	C8—C9—C10	119.85 (9)
C1B—C2B—H2BA	120.8	C8—C9—H9A	120.1
C2B—C3B—C4B	123.20 (9)	C10—C9—H9A	120.1
C2B—C3B—N1B	118.36 (8)	N3—C10—C11	120.35 (9)
C4B—C3B—N1B	118.42 (9)	N3—C10—C9	122.38 (9)
C4A—C3A—C2A	123.18 (9)	C11—C10—C9	117.27 (9)
C4A—C3A—N1A	118.57 (8)	C12—C11—C10	119.88 (9)
C2A—C3A—N1A	118.23 (9)	C12—C11—H11A	120.1
C3A—C4A—C5A	118.16 (9)	C10—C11—H11A	120.1
C3A—C4A—H4AA	120.9	N2—C12—C11	121.19 (9)
C5A—C4A—H4AA	120.9	N2—C12—H12A	119.4
C3B—C4B—C5B	117.67 (9)	C11—C12—H12A	119.4
			
C6A—C1A—C2A—C3A	−0.39 (14)	C4A—C5A—C6A—C1A	−0.97 (14)
C6B—C1B—C2B—C3B	0.50 (14)	C4A—C5A—C6A—C7A	178.78 (9)
C1B—C2B—C3B—C4B	1.38 (15)	C2B—C1B—C6B—C5B	−1.98 (14)
C1B—C2B—C3B—N1B	−176.79 (9)	C2B—C1B—C6B—C7B	176.30 (9)
O2B—N1B—C3B—C2B	−175.56 (9)	C4B—C5B—C6B—C1B	1.65 (14)
O1B—N1B—C3B—C2B	5.40 (14)	C4B—C5B—C6B—C7B	−176.66 (9)
O2B—N1B—C3B—C4B	6.18 (14)	C1A—C6A—C7A—O4A	−4.01 (14)
O1B—N1B—C3B—C4B	−172.86 (9)	C5A—C6A—C7A—O4A	176.24 (9)
C1A—C2A—C3A—C4A	−1.18 (15)	C1A—C6A—C7A—O3A	175.76 (8)
C1A—C2A—C3A—N1A	177.40 (8)	C5A—C6A—C7A—O3A	−3.99 (13)
O2A—N1A—C3A—C4A	3.87 (13)	C1B—C6B—C7B—O4B	170.01 (9)
O1A—N1A—C3A—C4A	−176.87 (9)	C5B—C6B—C7B—O4B	−11.69 (14)
O2A—N1A—C3A—C2A	−174.77 (9)	C1B—C6B—C7B—O3B	−11.13 (13)
O1A—N1A—C3A—C2A	4.48 (13)	C5B—C6B—C7B—O3B	167.17 (9)
C2A—C3A—C4A—C5A	1.64 (15)	C12—N2—C8—C9	−1.21 (16)
N1A—C3A—C4A—C5A	−176.93 (8)	N2—C8—C9—C10	1.08 (16)
C2B—C3B—C4B—C5B	−1.70 (15)	C8—C9—C10—N3	−179.88 (10)
N1B—C3B—C4B—C5B	176.47 (9)	C8—C9—C10—C11	−0.23 (15)
C3A—C4A—C5A—C6A	−0.53 (14)	N3—C10—C11—C12	179.16 (10)
C3B—C4B—C5B—C6B	0.15 (14)	C9—C10—C11—C12	−0.50 (15)
C2A—C1A—C6A—C5A	1.44 (14)	C8—N2—C12—C11	0.45 (16)
C2A—C1A—C6A—C7A	−178.30 (9)	C10—C11—C12—N2	0.41 (16)

### Hydrogen-bond geometry (Å, °)

**Table:**

*D*—H···*A*	*D*—H	H···*A*	*D*···*A*	*D*—H···*A*
O3A—H1O3···O3B^i^	0.82	1.63	2.4457 (11)	170
N3—H3A···O3B^ii^	0.86	2.14	2.9977 (12)	172
N3—H3B···O4B^i^	0.86	2.07	2.8758 (12)	155
N2—H1N2···O4A^iii^	0.85 (1)	1.99 (1)	2.7726 (12)	153 (1)
C2B—H2BA···O1B^iv^	0.93	2.52	3.2187 (13)	133
C8—H8A···O3A^v^	0.93	2.56	3.4565 (13)	161
C12—H12A···O1A^vi^	0.93	2.55	3.4427 (13)	162

Symmetry codes: (i) −*x*+1, −*y*+2, −*z*+1; (ii) −*x*+2, −*y*+2, −*z*+1; (iii) *x*+1, *y*−1, *z*; (iv) −*x*+3, −*y*+1, −*z*+2; (v) *x*, *y*−1, *z*; (vi) −*x*+2, −*y*+1, −*z*+1.
